# Targeting cancer with precision: strategical insights into TCR-engineered T cell therapies

**DOI:** 10.7150/thno.104594

**Published:** 2025-01-01

**Authors:** Pei Lin, Yunfan Lin, Zizhao Mai, Yucheng Zheng, Jiarong Zheng, Zihao Zhou, Xinyuan Zhao, Li Cui

**Affiliations:** 1 Stomatological Hospital, School of Stomatology, Southern Medical University, Guangzhou, 510280, Guangdong, China.; 2 Department of Dentistry, The First Affiliated Hospital, Sun Yat-Sen University, Guangzhou, 510080, China.; 3 School of Dentistry, University of California, Los Angeles, Los Angeles, 90095, CA, USA.

**Keywords:** T cell receptor-engineered T cell therapy, screening strategy, cancer immunotherapy

## Abstract

T cell receptor-engineered T (TCR-T) cell therapies are at the forefront of cancer immunotherapy, offering a transformative approach that significantly enhances the ability of T cells to recognize and eliminate cancer cells. This innovative method involves genetically modifying TCRs to increase their affinity for tumor-specific antigens. While these enhancements improve the ability of T cells to recognize and bind to antigens on cancer cells, rigorous assessment of specificity remains crucial to ensure safety and targeted responses. This dual focus on affinity and specificity holds significant promise for the treatment of solid tumors, enabling precise and efficient cancer cell recognition. Despite rapid advancements in TCR engineering and notable progress in TCR screening technologies, as evidenced by the growing number of specific TCRs entering clinical trials, several technical and clinical challenges remain. These challenges primarily pertain to the specificity, affinity, and safety of engineered TCRs. Moreover, the accurate identification and selection of TCRs that are both effective and safe are essential for the success of TCR-T cell therapies in cancer treatment. This review provides a comprehensive examination of the theoretical foundations of TCR therapy, explores strategies for screening specific TCRs and antigens, and highlights the ongoing challenges in this evolving therapeutic landscape.

## Introduction

**Figure 1:**
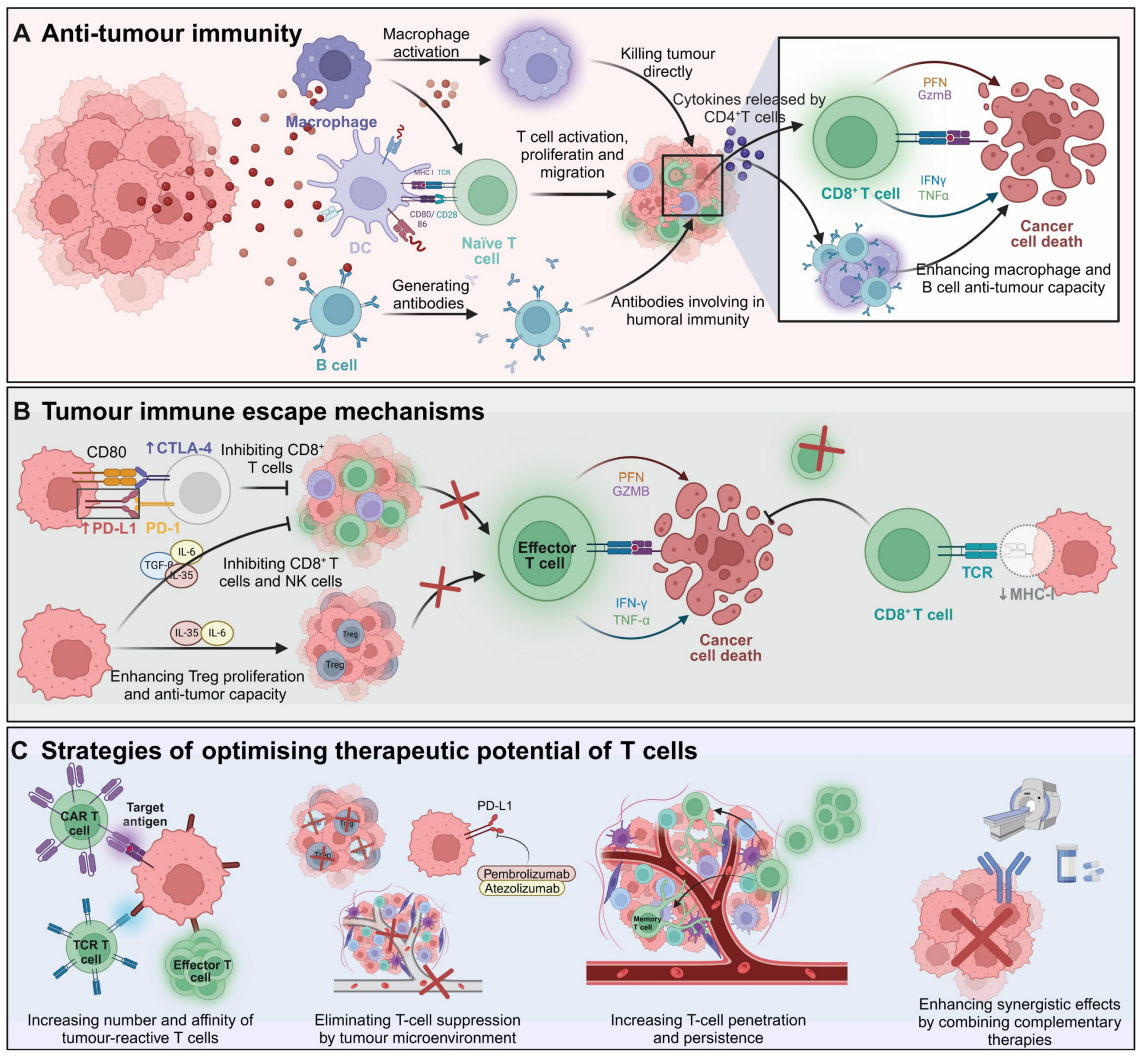
** Overview of T-cell immunity against tumors. A)** Tumor antigens are recognized by DCs, which activate naïve T cells and stimulate the generation of antigen-specific cytotoxic CD8^+^ T cells and helper CD4^+^ T cells. CD8^+^ T cells release PFN and GZMB to induce cancer cell apoptosis. CD4^+^ T cells secrete cytokines such as IFN-γ and TNF-α, enhancing CD8^+^ T cell cytotoxicity and promoting macrophage activation and B cell-mediated humoral immunity. **B)** Tumors evade immune surveillance through multiple mechanisms. They upregulate inhibitory molecules like PD-L1, which engage co-inhibitory receptors such as CTLA-4 on CD8^+^ T cells, thereby suppressing their effector functions. Additionally, tumors downregulate MHC class I molecules, reducing the ability of CD8^+^ T cells to recognize tumor antigens via TCR, leading to impaired recognition and elimination of cancer cells. Tumor-secreted cytokines, including IL-6, TGF-β, and IL-10, further inhibit the activity of CD8^+^ T cells and NK cells. These cytokines, along with IL-35, also promote the proliferation of Tregs, which play a crucial role in suppressing anti-tumor immune responses, further enabling tumor immune evasion.** C)** Strategies include CAR T cells and TCR-engineered T cells to enhance the number and affinity of tumor-reactive T cells. Immune checkpoint inhibitors such as pembrolizumab and atezolizumab restore T cell activity by counteracting the immunosuppressive TME. Additional approaches focus on improving T cell infiltration, persistence, and combining therapies for synergistic effects against cancer. The images in the figures were created using BioRender (https://www.biorender.com/).

T cells, a crucial subset of lymphocytes, exhibit intrinsic anti-tumor properties and are central to the immune defense against cancer. The anti-cancer response is mediated through a synergistic action of cytotoxic T cells (CD8^+^) and helper T cells (CD4^+^). CD8^+^ T cells target and eradicate malignant cells and proliferate rapidly upon encountering specific tumor antigens, thereby significantly contributing to the immune counterattack against cancer. Simultaneously, CD4^+^ T cells play a critical role in shaping adaptive immune responses and enhance these responses by activating CD8^+^ T cells 1, 2 (Figure 1A). Nevertheless, the effectiveness of this immune mechanism is often undermined by tumor strategies that evade immune detection, involving limited antigen presentation, immunosuppression, and the induction of T cell exhaustion 3, 4 (Figure 1B). These challenges underscore the imperative to develop strategies that enhance the therapeutic efficacy of T cells. Such strategies may include augmenting CD8^+^ T cell populations, boosting their antigen recognition capabilities, reducing factors that lead to exhaustion or suppression, and optimizing the supportive role of CD4^+^ T cells while curbing the generation of immunosuppressive regulatory T cells (Tregs) (Figure 1C).

Advancements in basic science have catalyzed the development of novel therapeutic approaches based on T cell-mediated immune responses. Checkpoint blockade therapy, for example, enhances T cell activity against tumors by blocking inhibitory pathways 2. Similarly, cellular immunotherapy has progressed significantly through TCR engineering, which improves T cell specificity by modifying their TCRs to better recognize specific tumor antigens, thus offering a promising strategy for personalized cancer immunotherapy 1, 5. Notably, TCR-T cell therapy offers distinct advantages over other cellular therapies, such as dendritic cell (DC) vaccines, CAR-T, and tumor-infiltrating lymphocyte (TIL) therapies, due to its unique capacity to target intracellular antigens presented by major histocompatibility complex (MHC) molecules. In contrast to CAR-T cells, which are limited to recognizing surface antigens via engineered receptors, TCR-T cells can engage a broader range of tumor-associated antigens, including those derived from intracellular proteins 6. This enhances their applicability to a wider spectrum of cancers, particularly solid tumors, where intracellular antigens are more common. Moreover, compared to TIL therapy, which requires the expansion of tumor-reactive T cells from patient biopsies—a process that can be lengthy and inefficient—TCR-T cells can be rapidly engineered to target specific antigens, streamlining the treatment process 7. While DC vaccines aim to prime the immune system by presenting antigens to T cells, their efficacy is often limited by the patient's existing immune landscape 8. In contrast, TCR-T cells provide a direct and potent antitumor response by engineering T cells with predefined specificity and affinity for tumor antigens. These factors make TCR-T cell therapy a versatile and powerful approach in the context of cancer immunotherapy, offering advantages in targeting a wider array of tumors with greater precision 9.

This review highlights several critical aspects of TCR-T cell therapy. First, we critically evaluate the current strategies for TCR screening, focusing on the methods used to identify and select tumor-specific TCRs with optimal therapeutic potential. Second, we systematically summarize the key clinical trials and preclinical studies of TCR-T therapy, providing an overview of its progress and therapeutic efficacy across various cancer types. Finally, we objectively analyze the challenges currently faced by TCR-T therapy, such as antigen selection, TCR affinity, and safety concerns, and propose potential strategies to overcome these barriers, aiming to enhance the clinical impact and applicability of TCR-T cell therapies in cancer treatment.

## Overview of TCR biology

### TCR structure and antigen recognition

The TCR complex, chiefly composed of TCRαβ or TCRγδ heterodimers alongside the CD3 signaling subunits, plays a pivotal role in T cell immune function. These TCR chains, transmembrane glycoproteins encoded by specific loci, couple non-covalently to form heterodimers with variable and constant immunoglobulin-like domains, a transmembrane domain, and a short cytoplasmic tail 10. Antigen recognition by the TCR involves three complementarity-determining regions (CDRs) on the α and β chains, interacting with peptide-MHC (pMHC) complexes 11. The CDR3 region is formed by DNA recombination involving juxtaposition of Vα and Jα segments for the α chain genes, and of Vβ, D, and Jβ segments for the β chain genes 12. The CDR1 and CDR2 regions, which exhibit limited diversity due to their germline-encoded nature, work in tandem with the highly variable CDR3 region, a key player in recognizing a wide range of antigens. The Vα CDR1 and CDR2 loops closely interact with the helices of the pMHC complex, whereas the Vβ CDR1 and CDR2 loops engage with the pMHC at the carboxy terminus of the bound peptide 13, 14. The TCR docks diagonally over the pMHC to initiate T cell activation, differentiation, and proliferation, ensuring consistent contact. This interaction is enhanced by CD4 and CD8 co-receptors 15. Importantly, the TCRα chain is positioned above the MHC-I α2-helix or MHC-II β1-helix, while the TCRβ chain docks above the α1-helix of both MHC-I and MHC-II, underlining the conserved docking topology crucial for effective immune responses 16, 17.

### TCR signaling and activation

**Figure 2:**
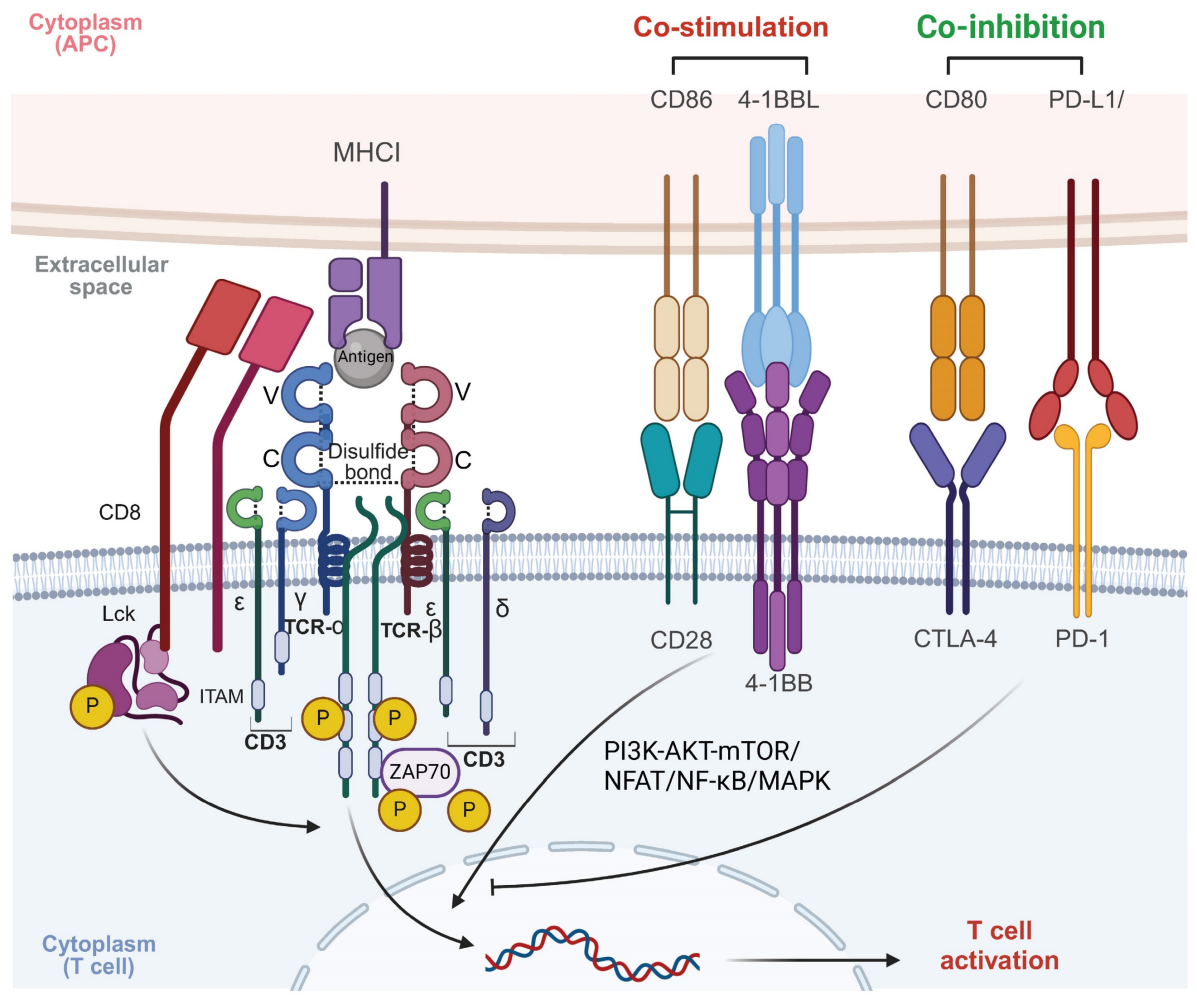
** Mechanism of T cell activation through TCR signaling.** The TCR complex recognizes antigenic peptides presented by MHC I molecules on APCs. CD8 co-receptor binds to MHC I, stabilizing the interaction. Upon antigen recognition, the ITAMs on CD3 are phosphorylated by the associated kinase LCK, leading to the recruitment and activation of ZAP70. This initiates downstream signaling cascades involving pathways such as PI3K-AKT-mTOR, NFAT, NF-κB, and MAPK, which ultimately result in T cell activation, proliferation, and effector functions. Co-stimulatory signals, provided by CD28 binding to CD86 and 4-1BBL binding to 4-1BB, are crucial for full T cell activation. These signals enhance the activation of downstream signaling pathways, promoting T cell survival, proliferation, and cytokine production. In contrast, co-inhibitory signals, mediated by CTLA-4 and PD-1 interacting with CD80 and PD-L1, respectively, dampen T cell activation. These inhibitory signals play a critical role in maintaining immune homeostasis and preventing overactivation, but in the context of cancer, they can contribute to immune evasion by tumors. Balancing co-stimulatory and co-inhibitory signals is essential for regulating T cell responses in both immunity and immunotherapy. The images in the figures were created using BioRender (https://www.biorender.com/).

Signal transduction in the TCR is primarily driven by the CD3 protein complex, due to the TCR's short intracellular domain. Hydrophobic interactions enable TCR binding to the CD3εδ, CD3εγ, and CD3ζζ dimers, forming the functional TCR-CD3 complex 18. Each CD3 subunit contains one to three immunoreceptor tyrosine-based activation motifs (ITAMs), essential for signal transduction. Upon TCR-pMHC interaction, a conformational shift and phosphorylation of ITAMs occur, initiating antigen-specific immune responses 19. T cell activation extends beyond TCR-pMHC binding, requiring interactions between various adhesion molecules on antigen-presenting cells (APCs) and T cells. These molecules deliver co-stimulatory signals crucial for stable and efficient T cell activation 20. After the primary TCR-pMHC signal, secondary signals are induced by co-stimulatory receptors like CD28 and ICOS, which interact with ligands such as B7-1 (CD80), B7-2 (CD86), ICOSL 21, 22. TNFSF/TNFRSF members, such as 4-1BB/CD137, are also critical co-stimulatory molecules, especially in CAR-T cell design 23. Notably, co-stimulatory molecules enhance TCR signals and significantly amplify T cell activation, proliferation, and differentiation by activating key signaling pathways such as PI3K-AKT-mTOR, NFAT, NF-κB, and MAPK 24. Additionally, co-inhibitory molecules on T cell surfaces, such as CTLA-4 and PD-1, transmit inhibitory signals that down-regulate or terminate T cell activation 25. These molecules are essential for maintaining immune homeostasis and self-tolerance, thus preventing autoimmune diseases and excessive immune responses 22 (Figure 2).

### TCR repertoire diversity

TCR diversity is primarily achieved through the combinational diversity of α and β chains and junctional variations at their joining segments 26. The α chain's variable region is encoded by V and J gene segments (TRAV and TRAJ), while the β chain includes V, D, and J segments (TRBV, TRBD, and TRBJ) 27. During thymic maturation, these segments undergo V-J or V-D-J recombination, generating diverse α and β chains that enhance TCR's ability to specifically recognize pMHC molecules 28. This process, coupled with random pairing of different α and β chains post-rearrangement, produces unique αβ TCR pairs with distinct antigen-binding capabilities 29. Junctional diversity, resulting from inaccuracies like frame shifts and nucleotide deletions during VDJ recombination, alters the amino acid composition of the V region, enhancing TCR specificity. Additionally, terminal deoxynucleotidyl transferase introduces random non-template nucleotides at the V(D)J junctions during rearrangement, greatly expanding the TCR repertoire 30. Consequently, VDJ recombination and non-template additions yield a theoretical TCR repertoire of approximately 2 × 10^19^ unique αβ TCR pairs 31. This extensive diversityenables recognition of a wide range of antigen peptides presented on pMHC.

## Tumor antigens and their relevance to TCR-T cell therapy

### Classification of tumor antigens

**Figure 3:**
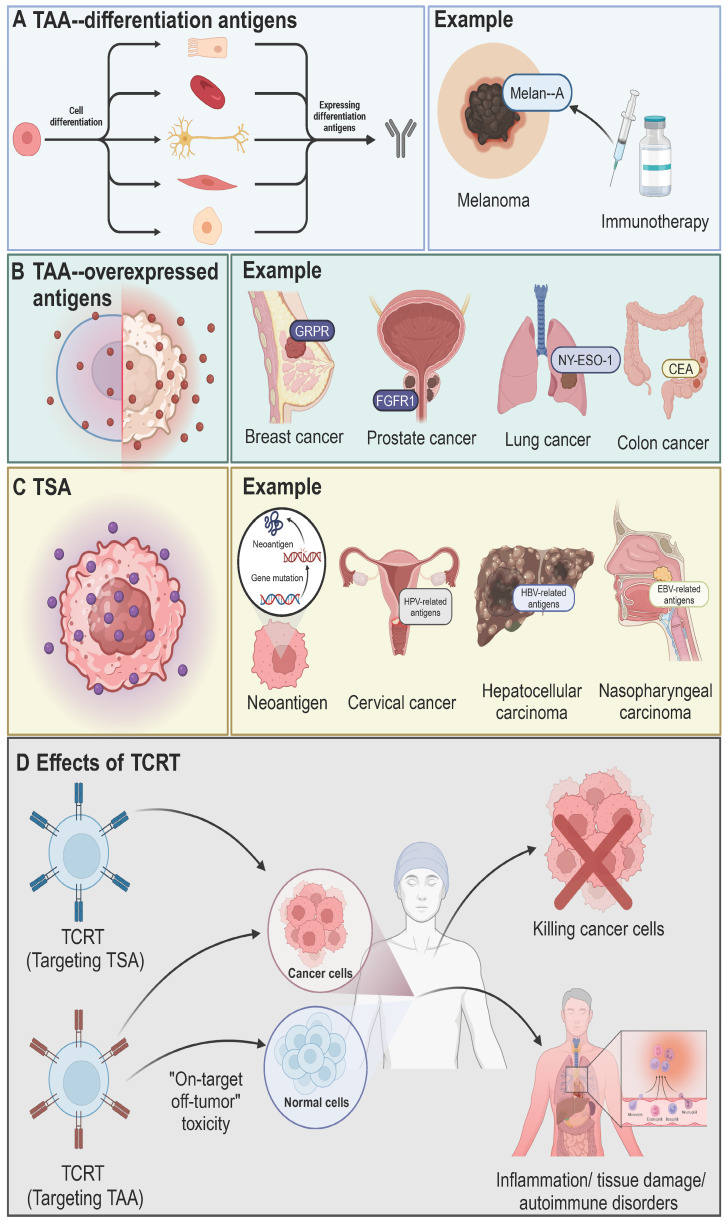
** Classification of tumor antigens. A)** Differentiation antigens are restricted to specific cell lineages and are expressed during cell differentiation. These antigens, such as Melan-A in melanoma, are highly immunogenic and can serve as targets for immunotherapy. Melan-A is selectively expressed in melanocytes and melanoma cells, and immunotherapies targeting this antigen have been developed to elicit specific anti-tumor immune responses. **B)** Overexpressed antigens are present at elevated levels in tumor cells compared to normal tissues. Examples include MUC1 in breast cancer, PSA in prostate cancer, NY-ESO-1 in lung cancer, and CEA in colon cancer. These antigens are frequently utilized as biomarkers and therapeutic targets due to their high expression levels in tumors, allowing for more selective cancer treatments and monitoring of disease progression.** C)** TSAs are derived from tumor-specific mutations or viral infections and are exclusively expressed by cancer cells. Neoantigens, arising from somatic mutations, are key targets for personalized immunotherapies. Viral antigens, such as those associated with HPV in cervical cancer, HBV in hepatocellular carcinoma, and EBV in nasopharyngeal carcinoma, also represent critical targets for immune-based interventions. TSAs play a central role in driving specific immune responses against tumors, minimizing off-target effects on normal tissues. **D)** TCR-T can target either TSAs or TAAs. Targeting TSAs is associated with a higher likelihood of selectively killing cancer cells without affecting normal cells due to the tumor-specific nature of these antigens. However, TCR-T targeting TAAs, which are overexpressed in tumors but also present in normal tissues, can result in "on-target, off-tumor" toxicity, leading to adverse effects such as tissue damage, inflammation, and autoimmune disorders. The images in the figures were created using BioRender (https://www.biorender.com/).

The therapeutic efficacy and safety of TCR-T cell therapy depend on the careful selection of tumor antigens. Successful tumor eradication relies on strong TCR-antigen binding, influenced by antigen processing and presentation. Tumor antigens are broadly classified into two categories: tumor-associated antigens (TAAs) and tumor-specific antigens (TSAs) 32 (Figure 3). TAAs are expressed in both normal and tumor cells and include differentiation and overexpressed antigens. Differentiation antigens, such as Melan-A, are linked to specific cell types and are used in cancer diagnosis and therapy 33. Overexpressed antigens, like FGFR1, NY-ESO-1, and carcinoembryonic antigen (CEA), show higher expression in tumors compared to normal tissues 34, 35. Cancer germline antigens (CGAs), a subset of TAAs, are aberrantly expressed in cancer cells and are naturally found only in germline cells of healthy tissues, such as testes 36. CGAs are promising targets for cancer treatments, with researched antigens including MAGE-A1, MAGE-A3, MAGE-A4, NY-ESO-1, PRAME, CT83, and SSX2, expressed in various solid tumors like melanoma and liver, lung, bladder, and neuroblastoma cancers 37. Nevertheless, targeting TAAs poses risks such as "on-target off-tumor" toxicity, leading to side effects like inflammation and tissue damage, and is further limited by restricted expression across cancer types, narrow HLA compatibility, and intra-tumoral heterogeneity 38, 39. TSAs, on the other hand, are exclusively expressed in tumor cells, reducing the risk of toxicity in normal tissues. High-affinity T cells that evade thymic negative selection can be isolated from patients or donors, making TSAs ideal targets 9. TSAs are classified into viral antigens, such as those from HPV and Epstein-Barr virus, and neoantigens, which arise from tumor-specific mutations 32, 40. Neoantigens are particularly promising due to their patient-specific nature, low toxicity, and ability to escape negative selection, making them a key focus of current immunotherapy research 41.

### Processing and presentation of different types of antigens

#### Processing and presentation of endogenous antigens

**Figure 4:**
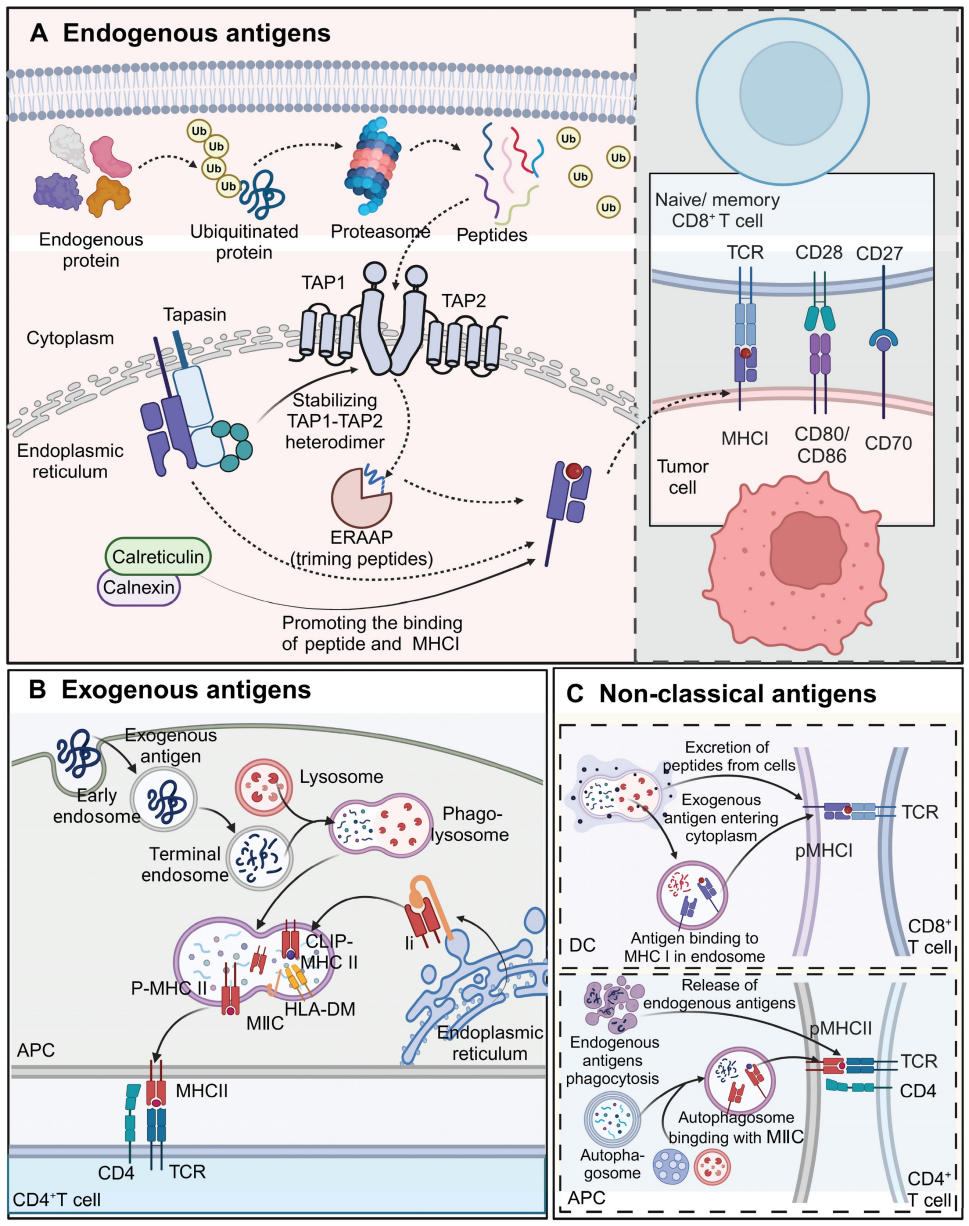
** Processing and presentation of different types of antigens. A)** Endogenous proteins are ubiquitinated and degraded into peptides by the proteasome. Peptides are transported by the TAP1-TAP2 complex into the endoplasmic reticulum, where they are trimmed by ERAAP and loaded onto MHC I molecules. Calreticulin and calnexin assist in peptide loading and stabilization of MHC I. Once the peptide-MHC I complex is formed, it is transported to the cell surface to be recognized by CD8^+^ T cells, promoting cytotoxic T cell responses against tumor cells expressing these antigens. Co-stimulatory molecules such as CD80/CD86 and CD70 further enhance T cell activation. **B)** Exogenous antigens are taken up by APCs via endocytosis or phagocytosis. These antigens are processed in lysosomes and loaded onto MHC II molecules within the MIIC compartment, facilitated by HLA-DM, which removes CLIP from the MHC II complex. The peptide-MHC II complex is transported to the cell surface, where it is recognized by CD4^+^ T cells. This interaction triggers helper T cell activation and subsequent immune responses. **C)** Exogenous antigens can also enter the cytoplasm of APCs and be cross-presented on MHC I molecules, enabling CD8^+^ T cell activation. Additionally, endogenous antigens can be processed through autophagy, with autophagosomes delivering cytoplasmic contents, including endogenous antigens, to the MIIC for loading onto MHC II. Both cross-presentation and autophagy-mediated presentation allow for the activation of distinct subsets of T cells, broadening immune recognition of tumor antigens. The images in the figures were created using BioRender (https://www.biorender.com/).

Endogenous antigens, including viral proteins, tumor antigens, and certain self-antigens, are processed via the MHC class I pathway and presented to CD8^+^ T cells. This process begins with the degradation of defective proteins by the ubiquitin-proteasome system 42. The resulting peptides are translocated into the ER lumen by the ATP-dependent transporter associated with antigen processing (TAP), composed of TAP1 and TAP2 subunits. Some peptides undergo further trimming by ER-associated aminopeptidase (ERAAP) to fit the MHC class I binding groove optimally 43, 44. The peptide-loading complex, consisting of TAP, tapasin, ERp57, and calreticulin or calnexin, facilitates peptide binding to MHC class I molecules 45. Tapasin stabilizes the TAP heterodimer and promotes peptide loading onto MHC-I molecules 46. The loaded MHC-I complexes are then transported to the cell surface for recognition by CD8^+^ T cells. Since TCR-T cell therapies typically target endogenous antigens and rely on CD8^+^ T cells, the MHC class I pathway is central to TCR-T cell recognition and tumor elimination 47 (Figure 4A).

#### Processing and presentation of exogenous antigens

Exogenous antigens, such as bacterial toxins, proteins, and pathogens, are processed via the MHC class II pathway and presented to CD4^+^ T cell. APCs, including DCs, B cells, macrophages, and thymic epithelial cells, internalize exogenous antigens through phagocytosis and endocytosis. Internalized proteins are degraded into peptides in phagolysosomes, which then merge with MHC class II compartments (MⅡCs) for peptide loading 48. Most MHC-II peptides are loaded through the invariant chain (Ii)-dependent pathway, where the Ii protein blocks the binding groove until hydrolysis generates class II-associated invariant chain peptide (CLIP), which is then displaced by HLA-DM, allowing antigenic peptide binding 49. These peptide-MHC-II complexes are transported to the APC surface for recognition by CD4^+^ T cells, a crucial step in immunotherapies targeting exogenous antigens (Figure 4B).

#### Non-classical antigen processing and presentation pathways

Cross-presentation enables the presentation of exogenous antigens via MHC class I to CD8^+^ T cells or endogenous antigens via MHC class II to CD4^+^ T cells. DCs play a primary role in cross-presentation 50. Mechanisms include the direct translocation of exogenous antigens into the cytoplasm or their escape from phagolysosomes for binding to MHC-I molecules, or the binding of lysosome-generated peptides to surface MHC-I molecules via exocytosis 51. This pathway is important for stimulating CD8^+^ T cells in response to vaccines and pathogens, enhancing antitumor immunity. Moreover, MHC-II molecules have been implicated in presenting endogenous antigens to CD4^+^ T cells, with autophagy potentially facilitating the cross-presentation of cytoplasmic and nuclear proteins 52 (Figure 4C).

## Challenges in tumor antigen identification and targeting

The therapeutic success of TCR-T cell therapy depends on the precise selection and effective binding of engineered TCRs to tumor antigens. While theoretically capable of targeting any tumor antigen, the selection of targets that ensure both safety and efficacy remains a significant challenge 53. Targeting antigens like viral antigens, neoantigens, and cancer-testis antigens, which are predominantly expressed in tumor cells and minimally in non-essential tissues, reduces the risk of damage to normal cells 54. However, TAAs, even with elevated expression in tumors, may cause autoimmune toxicity due to their presence in normal tissues. The balance between TCR affinity and antigen recognition is crucial; insufficient affinity risks inadequate tumor cell recognition, while excessively high affinity can lead to overactivation, immune toxicity, and severe adverse effects 55.

Tumor antigen heterogeneity, characterized by variability in antigen expression across different tumors or even within the same tumor, presents additional challenges for consistent therapeutic outcome. This phenotypic diversity, driven by genetic mutations and environmental factors, allows some tumor cells to evade targeted therapy, leading to potential resistance to treatments. Moreover, inter-individual differences, particularly polymorphisms in MHC molecules, can result in varying immune responses to the same tumor antigen, affecting the efficacy of TCR-T cell therapy across patients. Strategies to address this issue, such as targeting multiple antigens or promoting epitope spreading via genetic engineering, are being explored 56.

Tumor cells employ multiple strategies to undermine the efficacy of TCR-T cell therapy, such as reducing TCR-antigen binding, altering antigen presentation, upregulating immune checkpoint molecules, and fostering an immunosuppressive tumor microenvironment that inhibits T cell function 57, 58. Tumor cells can impair antigen presentation by reducing MHC class I expression, a phenomenon linked to mutations or epigenetic silencing of HLA genes. Additionally, defects in the antigen processing machinery pathway, crucial for MHC-antigen complex formation, can enable tumors to evade immune detection 59. The tumor microenvironment exacerbates immune evasion by promoting immunosuppressive conditions, such as cytokine release and hypoxia-induced lactic acid production, which impair TCR-T cell function 60, 61.

In addition, engineered TCR-T cells' inability to distinguish between cancerous and normal cells expressing the same antigens remains a significant challenge. This lack of specificity, particularly concerning TAAs, can lead to severe off-target effects in healthy tissues. For example, fatal cardiotoxicity has been observed in metastatic melanoma patients treated with TCR-T cells targeting MART-1 and MAGE-A3, likely due to antigen expression in cardiac tissues 57. Moreover, MART-1 and gp100-targeted TCR-T cells have been associated with ocular, cutaneous, and auditory toxicities due to TAA expression in melanocytes 62. Off-target effects, characterized by unintended damage to non-target tissues, and cross-reactivity, where TCR-T cells recognize structurally similar antigens in unrelated tissues, such as MAGE-A3 TCRs binding to epitopes from MAGE-A12 or the TITIN protein, complicate the application of TCR-T cell therapies 63. To mitigate these risks, approaches such as optimizing TCR structure and expression, as well as utilizing gene-editing technologies like CRISPR-Cas9 for antigen refinement, are under development. Close monitoring and effective management of adverse events are critical to ensuring the safety and efficacy of TCR-T cell therapies.

## TCR screening approaches for optimizing TCR-T cell therapy

TCR screening is critical for the success of TCR-T cell therapy, particularly in personalized cancer immunotherapy. This process involves selecting TCRs with the appropriate specificity and affinity for tumor antigens to ensure precise targeting of cancer cells while minimizing off-target effects on normal tissues. Effective TCR screening is essential for developing tailored TCR-engineered therapies, improving therapeutic efficacy and reducing the risk of immune-related adverse events. Additionally, this approach facilitates the identification of novel tumor-specific antigens, expanding the potential applications of immunotherapy in cancer treatment 64.

### Cell-based screening

**Figure 5:**
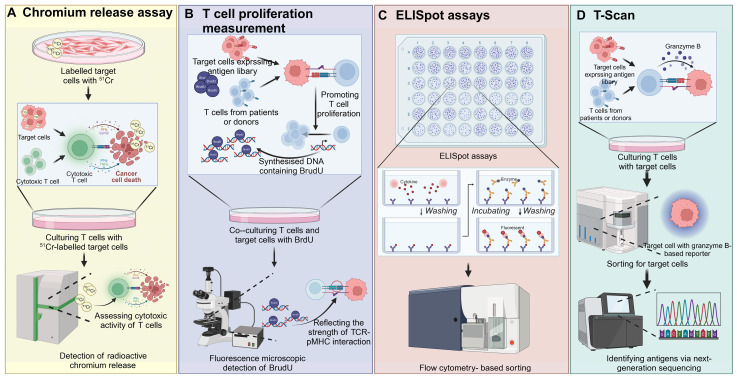
** Cell-based TCR screening strategies. A)** Target cells are labeled with radioactive chromium (^51^Cr) and co-cultured with cytotoxic T cells. Upon recognition and lysis of target cells by T cells, chromium is released into the supernatant. The level of chromium release is measured to assess T cell cytotoxicity, providing a quantitative evaluation of the T cell-mediated killing of cancer cells. **B)** T cells from patients or donors are co-cultured with target cells expressing a library of antigens. The proliferation of T cells is monitored by the incorporation of BrdU into newly synthesized DNA during cell division. The level of BrdU incorporation, detected through fluorescence microscopy, reflects the strength of the TCR-pMHC interaction, providing insights into T cell responses against specific antigens. **C)** ELISpot is used to detect cytokine secretion from individual T cells in response to target cells. T cells are co-cultured with target cells, and cytokine release is captured on a membrane pre-coated with specific antibodies. After incubation and washing, spots representing single T cell cytokine release are visualized. Flow cytometry-based sorting can then be used to isolate specific subsets of T cells for further analysis.** D)** In T-Scan, T cells are cultured with target cells expressing an antigen library. Granzyme B release upon target cell recognition is measured, serving as an indicator of cytotoxic activity. Target cells are sorted based on granzyme B reporter expression, and next-generation sequencing is used to identify the antigen that triggered the T cell response, enabling the identification of novel antigens. The images in the figures were created using BioRender (https://www.biorender.com/).

Cell-based screening strategies are indispensable in immunology for evaluating TCR-pMHC interactions and assessing the functional capacity of T cells after exposure to APCs expressing pMHC complexes. Several key techniques are utilized, each providing unique insights into T cell function 65. T cell proliferation assays quantify the expansion of T cells in response to antigenic stimulation, often using [3H]-thymidine incorporation to measure DNA synthesis or CFSE dilution to track cell division. These assays provide a direct measure of T cell activation induced by TCR engagement with specific pMHC complexes, indicating the strength of the interaction and the capacity of T cells to proliferate in response to antigen 66. Chromium release assays assess T cell cytotoxicity by labeling target cells with the radioactive isotope ^51^Cr. Upon T cell-mediated lysis, the release of ^51^Cr into the culture medium is measured, with higher levels indicating stronger cytolytic activity 67. ELISpot assays are highly sensitive and allow for the detection of cytokine secretion at the single-cell level. In this assay, T cells are incubated on antibody-coated plates, where cytokines such as IFN-γ secreted in response to antigen stimulation are captured. A secondary antibody, linked to an enzyme, produces visible spots after binding to the captured cytokine, providing a quantitative measure of antigen-specific T cell responses 68. Peptide-based ELISpot assays were used to screen for potential glioma neoepitopes in MHC-humanized mice, leading to the identification of MHCII-restricted CICR215W/Q as immunogenic. Following vaccination, TCR sequencing from neoepitope-specific T-cell lines enabled the discovery of CICR215W-specific TCRs. Adoptive intraventricular transfer of these TCR-transgenic T cells showed antitumor efficacy in a glioma model 69. Similarly, using exome sequencing data from TCGA, shared neoantigen peptides were predicted, and ELISpot assays were employed to screen for CD8^+^ T cells reactive to the mutated FGFR3^Y373C^ peptide in HLA-A*02:06 donors. FGFR3^Y373C^-specific TCRs were identified and expressed in engineered T cells, which selectively recognized the mutated peptide and exhibited cytotoxic activity 70. Intracellular cytokine staining involves the fixation and permeabilization of activated T cells, allowing antibodies to access intracellular cytokines produced during TCR engagement. T cells are stained with fluorescently labeled antibodies targeting cytokines such as IFN-γ, TNF-α, or IL-2, and analyzed via flow cytometry to quantify the proportion of cytokine-producing T cells. To enhance detection, inhibitors such as Brefeldin A or Monensin are used to block cytokine secretion, ensuring their accumulation within the cells for accurate measurement 71. T-Scan technology is a high-throughput, genome-wide approach that identifies antigens recognized by T cells. Using MHC-I molecules to display peptides from pathogen or human protein libraries, this system employs a granzyme B-based reporter to detect T cell-mediated killing. Target cells expressing specific antigens that induce T cell cytotoxicity are isolated, and the corresponding antigens are identified using next-generation sequencing (NGS). Unlike traditional methods that focus on TCR-pMHC binding affinity, T-Scan emphasizes T cells' natural cytolytic activity, effectively identifying both known and novel peptide epitopes and providing a comprehensive map of TCR specificity 62 (Figure 5).

### Yeast / phage / mammalian cell display library

The complexity of pMHC-TCR interactions is largely driven by MHC polymorphism and the extensive diversity of peptides and TCRs. The integration of traditional biochemical methods with combinatorial biology techniques, particularly through the use of peptide or protein libraries, has proven to be an effective strategy for elucidating these complex biological interactions 72. Yeast display of pHLA libraries is a scalable method for identifying TCR ligands, enabling the screening of millions to billions of epitopes. These libraries, comprising approximately 10^7^ to 10^9^ unique clones, offer benefits such as low reagent costs and rapid cycle times of 2-3 weeks for library generation and selection 73. In this system, the pMHC complex is anchored to the yeast surface protein AGA2P, with the TCR featuring Vα and Vβ domains connected by a GlySer linker attached to the C-terminus of AGA2P 72. This configuration ensures accurate orientation and stable presentation of TCR-pMHC interactions. The use of epitope tags and specific probes allows for monitoring of protein expression and functionality, with flow cytometry providing precise antigen screening 72. Notably, yeast-display libraries were utilized to identify antigens for "orphan" TCRs on tumor-infiltrating lymphocytes from colorectal adenocarcinoma, revealing that four TIL-derived TCRs selectively bound peptides from a diverse pHLA-A∗02:01 library. Three TCRs targeted non-mutated self-antigens, including a shared antigen derived from U2AF2, demonstrating yeast display's effectiveness in uncovering TCR specificities and identifying tumor antigens 74. Interestingly, the yeast agglutination mediated TCR antigen discovery system (YAMTAD) enhances the screening of TCR-pMHC interactions, achieving rapid, high-throughput identification of TCRs and their cognate antigens without the need for purification. Demonstrating high sensitivity and specificity, YAMTAD supports personalized immunotherapy by facilitating the enrichment of high-affinity TCR-pMHC interactions, offering a practical solution for library-on-library screening 75.

Phage display is employed to create diverse, functional TCR libraries by presenting human TCRs on the surface of M13 phages, utilizing a stabilized TCR heterodimer with an added disulfide bond for increased stability and compatibility with various TCR sequences and MHC molecules 76, 77. For example, phage display facilitated the development of a TCR-like antibody, 2D2, targeting the NY-ESO-1/HLA-A2 complex, essential for recognizing intracellular cancer targets. This technology enabled the creation of CAR-T cells that effectively targeted and reduced tumor growth in melanoma and breast cancer models, showcasing its potential to expand antigen recognition in immunotherapy 78. Additionally, a novel phage display screening strategy was developed to enable fast and easy selection of thermostabilized proteins. Random mutant scTCR phage libraries were prepared in E. coli overexpressing the ectoplasmid chaperone protein FkpA, which screened for and isolated heat-stable scTCR (mutant) variants. Then the tumour-specific reactivity of this scTCR was demonstrated by surface plasmon resonance technique 79.

Additionally, the Chinese Hamster Ovary (CHO) display has been utilized for the affinity engineering of TCRs, specifically in the development of bispecific TCR-based therapies. This system enables stable integration of TCR molecules, allowing for the efficient selection of high-affinity variants targeting cancer-specific antigens like PRAME. This approach demonstrates significant therapeutic potential, notably in eliciting strong immune responses against tumor cells 80. Similarly, mammalian cell display was used to engineer the CMV-specific TCR RA14 for high-affinity and soluble expression. By screening CDR3 libraries, clones with enhanced pMHC affinity were identified. These high-affinity TCRs retained peptide specificity and activation in Jurkat T cells. Soluble expression was optimized via Fc fusion, disulfide bonding, and glycosylation site disruption, yielding a variant with 50 nM pMHC affinity and specific cell staining 81.

### pMHC tetramer-based screening

pMHC multimers are essential tools for detecting antigen-specific T cells, as they replicate the natural presentation of peptides by MHC molecules on cell surfaces that T cells recognize. When conjugated with fluorochromes, these multimers allow for T cell detection via conventional flow cytometry 82. pMHC tetramer-based screening with DNA barcoding improves the detection of rare antigen-specific T cells, offering higher sensitivity than conventional methods. By using photocleavable linkers, DNA-barcoded tetramers enable efficient T cell staining and barcode recovery. This approach allows for simultaneous detection of multiple antigen-specific T cell populations, facilitating precise monitoring of immune responses in cancer and vaccine studies 83. Similarly, pMHC tetramer-based screening enhanced with magnetic nanoparticles and barcoded DNA linkers efficiently isolates neoantigen-specific CD8^+^ T cells. This method shows superior recovery over traditional approaches and is valuable for monitoring immune responses in cancer immunotherapy 84.

Notably, pMHC tetramer-based screening revealed that patients with metastatic urothelial carcinoma who responded to PD-L1 blockade exhibited an expansion of neoantigen-reactive CD8^+^ T cell (NART) populations post-treatment. NARTs displaying a PD1^+^ Ki67^+^ effector phenotype and high CD39 expression were associated with disease control, distinguishing them from bystander T cells during immune checkpoint blockade. Among 37 predicted neoantigens, tetramer staining identified potential HCC-dominant neoantigens restricted by HLA-A11:01, HLA-A24:02, or HLA-A02:01. Specifically, HLA-A24:02-restricted FYAFSCYYDL and HLA-A*02:01-restricted WVWCMSPTI demonstrated strong immunogenicity, which was confirmed using the Co-HA system. This method validated neoantigen-specific T cells and their antitumor efficacy, highlighting the role of tetramer-based screening in verifying clinically relevant neoantigens 85. Furthermore, Tetramer-Associated T-Cell Receptor Sequencing (TetTCR-Seq) enables high-throughput linking of TCR sequences to their cognate antigens using DNA-barcoded pMHC tetramers. This method allows for the rapid identification of antigen-specific TCRs, including those recognizing cancer neoantigens without cross-reactivity to wild-type antigens 86. Advances in using pMHC multimers attached to rare metal ions for detection via time-of-flight mass spectrometry (CyTOF) overcome the limitations of flow cytometry and enable more complex cell analysis. Using CyTOF and metal-labeled probes, CD8^+^ T cells were analyzed for surface markers, cytokines, and antigen specificity via peptide-MHC tetramers. This revealed extensive phenotypic and functional diversity within CD8^+^ T cells, including a nearly combinatorial pattern of cytokine expression despite sharing antigen specificity. Principal component analysis highlighted the continuous nature of CD8^+^ T cell differentiation and the complexity of the compartment, underscoring their flexibility in immune responses to pathogens 87. Additionally, a method combining mass cytometry with combinatorial peptide-MHC tetramer staining enables rapid identification and characterization of T cells specific for numerous epitopes. Screening up to 10^9^ peptide-MHC tetramers in a single blood sample while analyzing 23 additional markers revealed six rotavirus-specific T cell epitopes in HLA-A*0201 individuals. T cells targeting the VP3 protein showed distinct phenotypes and were abundant in intestinal epithelium, demonstrating the utility of this approach for analyzing T cell responses to infections or vaccines 88.

### Trogocytosis

**Figure 6:**
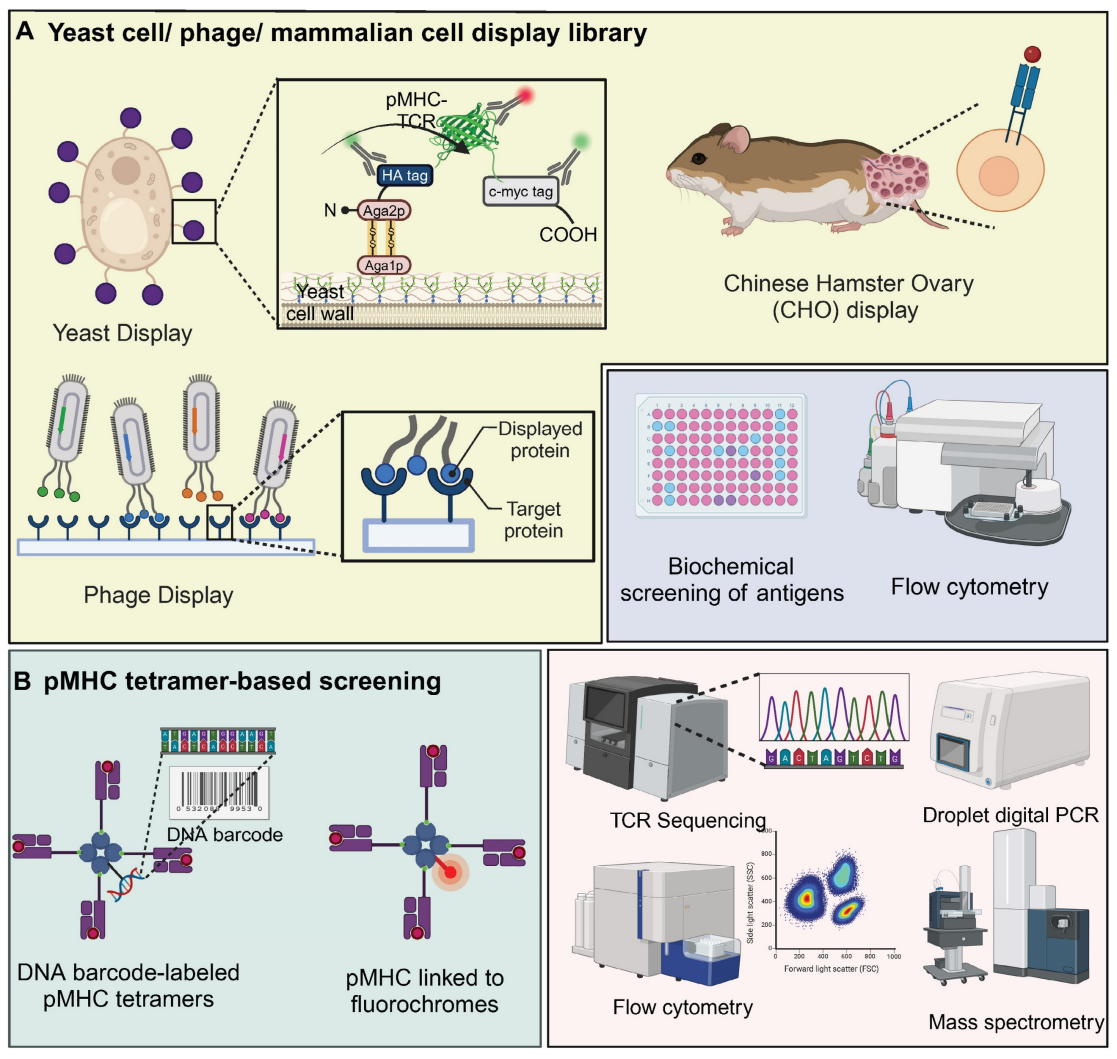
** TCR screening using display libraries and pMHC tetramer-based assays. A)** TCR libraries displayed on yeast, phages, and mammalian cells enable the high-throughput screening of antigen specificity. Yeast display incorporates TCRs anchored to the cell wall via Aga2p, facilitating the screening against labeled pMHC complexes. Phage display vectors present TCRs on capsid proteins, allowing for rapid affinity maturation through successive biopanning rounds. CHO cells expressing TCR-pMHC constructs provide a mammalian context for functional analyses. The resulting interactions are analyzed via biochemical screening, flow cytometry, and other downstream assays to identify TCRs with desired antigen specificities.** B)** pMHC tetramer-based screening utilizes DNA barcode-labeled pMHC tetramers linked to fluorochromes, facilitating simultaneous analysis of multiple TCR-pMHC interactions through flow cytometry. The barcodes allow for precise identification and quantification of TCR engagement, while TCR sequencing and droplet digital PCR provide detailed insights into the TCR repertoire. Mass spectrometry complements these analyses by identifying specific peptide-MHC interactions, enhancing the understanding of TCR specificity and affinity. The images in the figures were created using BioRender (https://www.biorender.com/).

Trogocytosis is a process in which immune cells, particularly T cells, acquire membrane proteins such as MHC molecules from APCs during intercellular interactions. This bidirectional transfer of surface molecules occurs at the immunological synapse, where T cells recognize antigens presented by MHC molecules on APCs via their TCRs 89. During synapse formation, T cells internalize fragments of the APC membrane, including MHC-peptide complexes, through vesicular transport mechanisms, while APCs can simultaneously acquire T cell-specific molecules, including TCRs. This exchange allows APCs to retain a molecular "fingerprint" of their interaction with antigen-specific T cells, thereby facilitating the identification of matching TCRs 90. In TCR screening applications, trogocytosis enables the isolation of antigen-specific TCRs by tracking the acquired MHC-peptide complexes. This process is valuable for the identification of TCRs with high antigen specificity, advancing TCR-based immunotherapies and vaccine development. For example, trogocytosis was employed to identify tumor-specific TCRs from the exhausted T cell pool in post-allogeneic hematopoietic stem cell transplant patients. Although TAA-specific T cells were present in 90% of patients, they exhibited an exhaustion phenotype with limited effector function. Using trogocytosis and ligandome-on-chip technology, it was possible to isolate TCRs, including those recognizing novel acute myeloid leukemia antigens, highlighting the potential for TCR-based cancer immunotherapy 91 (Figure 6B).

### Microfluidic technology in TCR screening

Microfluidic technology, with its ability to precisely control small fluid volumes, plays a critical role in TCR screening by enabling high-throughput, single-cell analysis in highly controlled microenvironments. Through microfluidic chips, individual T cells are isolated and exposed to peptide-MHC complexes, allowing for the real-time observation of antigen-specific T cell activation 92. This approach integrates advanced detection methods, such as fluorescence or barcode-based systems, to assess TCR-antigen interactions with high sensitivity and specificity. Microfluidics significantly enhances screening efficiency by enabling rapid, parallel analysis of TCR functionality, facilitating the identification of therapeutically relevant TCRs. For instance, droplet microfluidics enables rapid, multiplexed functional screening of single TCR T cells by monitoring real-time activation upon tumor cell recognition. The platform features clone tracking and a highly specific sorting system, with 100% accuracy confirmed by single-cell RT-PCR and TCR sequencing. This microfluidic-based approach streamlines TCR screening, accelerating the development of effective T cell therapies 93. Similarly, a high-throughput microfluidic approach was developed to identify TCRs with high functional avidity from diverse human T cell repertoires. By generating libraries of full-length TCRαβ clones from millions of primary T cells, expressed in Jurkat cells, this method enabled repeated screening. Over 2.9 million TCRαβ clonotypes were captured, including rare viral-antigen-reactive and tumor-specific TCRs from melanoma patient samples, which mediated effective tumor cell killing 94. Additionally, microfluidic technology isolates peptide-specific CD8^+^ T cells using magnetic nanoparticles functionalized with peptide-MHC tetramers. This platform captures antigen-specific T cells, preserving the link between peptide and TCR gene identity, and allows simultaneous analysis of multiple peptides with just 100,000 cells. It offers 1,000-fold greater sensitivity than bulk methods, enabling the detection of rare viral antigen-specific TCRs 95. Notably, a novel microfluidic-based approach identifies and recovers potent T cell clones by measuring cellular avidity between T cells and tumor cells. This method probes up to 10,000 T cell-tumor interactions per run and recovers highly avid T cells with 100% purity in 30 minutes. The recovered T cells retain cytotoxicity, activation, and avidity markers upon re-exposure to tumor cells, accelerating the selection process for therapeutic T cells and advancing precision cancer immunotherapy 96. Likewise, a novel droplet microfluidic platform enables high-throughput screening of pMHC and TCR pairs with high sensitivity and minimal background noise. By integrating DNA barcoding technology, antigen-loaded cells and reporter cells can be labeled to identify pMHC-TCR specificity. Coupled with next-generation sequencing, this platform precisely maps pMHC-TCR interactions, offering potential for cross-reactivity and off-target screening in clinical TCR applications 97.

### Single-cell sequencing in TCR screening

Single-cell sequencing is a critical method for the high-throughput isolation of tumor-specific TCR encoding genes, essential for elucidating immune responses against cancer. This technique involves isolating individual T cells using flow cytometry or microfluidics, followed by cell lysis and reverse transcription to convert RNA into cDNA that includes TCR sequences 98. Targeted sequencing, often facilitated by RNA baits that specifically hybridize to V and J segments of the TCRα and TCRβ chains, allows for selective enrichment and sequencing of these critical TCR components. This approach preserves the native TCR pairings and enables a comprehensive analysis of the TCR repertoire across thousands of T cells, providing detailed insights into the diversity and functionality of immune responses at the cellular level. Such precise capture of TCR sequences is fundamental for assessing antigen specificity and T cell diversity within the TME 12.

For instance, SEQTR, a high-throughput approach, enhances the sensitivity, reproducibility, and accuracy of analyzing human and mouse TCR repertoires, surpassing traditional assays in capturing the complexity of blood and tumor TCR landscapes. This method, combined with a novel TCR cloning strategy, streamlines the discovery, screening, and engineering of tumor-specific TCRs, significantly advancing TCR repertoire analyses and accelerating the development of cellular therapies in clinical settings 99. Additionally, single-cell sequencing enables efficient isolation of neoantigen-specific TCRs by analyzing tumor-infiltrating T cells stimulated with neoantigen-loaded DCs. By sequencing TCRs and activation markers, this method identified 28 unique TCRs from melanoma and colorectal tumor samples, showing high reliability when identical sequences were detected in multiple cells. This high-throughput approach streamlines TCR identification for both research and clinical applications in cancer immunotherapy 98. Notably, by analyzing TILs co-cultured with APCs, single-cell sequencing identifies TCR sequences linked to high IFN-γ and IL-2 expression. This method streamlines the isolation of neoantigen-specific TCRs, enabling the transduction of donor T cells for improved neoantigen recognition in cancer therapy 100.

### Computational methods for TCR screening

Computational methods have become essential for the effective screening of TCR, enabling detailed characterization and prediction of TCR-antigen interactions at a significant scale. These approaches employ advanced algorithms to predict the binding affinity of TCRs with pMHC complexes, thereby streamlining the selection of potential therapeutic targets. Computational tools also analyze TCR repertoires from high-throughput sequencing data, interpreting complex sequence information to uncover diversity and clonality within a population. Such methodologies not only accelerate the identification of tumor-specific TCRs but also enhance our understanding of T cell mediated immunity. This supports the development of personalized T cell therapies and provides critical insights for the manipulation of immune responses in various pathologies. For instance, the TRUST algorithm was used to assemble TCR CDR3 regions from 9,700 tumor RNA-seq samples, while the iSMART methodology clustered similar TCRs to infer shared antigen specificity. Computational integration of multi-omics data facilitated the profiling of T cell behavior and the discovery of novel cancer antigens, enhancing the potential for targeted immune therapies in oncology 101. Addtionally, TABR-BERT, a deep learning model based on BERT, enhances the prediction of TCR-pMHC binding by capturing key information from TCR sequences, antigen epitopes, and their interactions. This approach shows improved performance in benchmark tests, especially for unseen epitopes, demonstrating its potential to refine TCR-based cancer immunotherapy development 102. Moreover, the TCR-ESM model, leveraging peptide embeddings from the evolutionary scale modeling protein language model, enhances TCR-pMHC binding predictions. Training on paired TCR data incorporating both CDR3α and CDR3β chain information yields superior performance compared to using only CDR3β, underscoring the contribution of both chains to specificity. This approach demonstrates generalizability across external datasets, spotlighting deep learning's potential to advance understanding of TCR specificity and interaction dynamics 103. TULIP, a transformer-based unsupervised language model, addresses the limitations of existing TCR-epitope binding prediction methods by utilizing incomplete datasets and unsupervised learning. This flexible model integrates various data qualities and has demonstrated reduced bias from sampling procedures common in supervised approaches 104.

Computational methods also play a critical role in enhancing the prediction of peptide-MHC binding interactions, providing crucial insights for immunological research and therapeutic development. NetMHCpan-2.0 predicts peptide-MHC class I interactions across species, including uncharacterized human and non-human MHC molecules. Trained on extensive data, it accurately identifies binding peptides, aiding immunological research across diverse populations 105. Similarly, NetMHCpan-4.1 and NetMHCIIpan-4.0 are web servers designed to predict peptide binding to MHC Class I and Class II molecules, respectively. These tools utilize advanced machine learning techniques to integrate binding affinity data and mass spectrometry-eluted ligands, enhancing the accuracy of predictions. This improvement in predictive capability is crucial for determining the specificity of T cell responses, significantly advancing TCR screening and immune response profiling 106. In addition, MHCSeqNet, a deep learning model, advances MHC binding prediction by employing neural network architectures akin to those used in natural language processing. This approach models amino acid sequences of MHC alleles and epitope peptides as sentences, enhancing performance across both binding affinity and ligand peptidome datasets. Its ability to generalize to novel MHC class I alleles and accept peptides of varying lengths makes it a robust tool for neoepitope screening in cancer vaccine development 107.

Furthermore, recent computational advancements have transformed the analysis of TCR repertoires, enabling precise pMHC-TCR clustering, pairing, and deorphanization. The GLIPH algorithm clusters TCRs by conserved CDR3 motifs and sequence similarity, enabling pMHC-TCR pairing and large-scale deorphanization across donors. Validated with *Mycobacterium tuberculosis*-reactive TCRs, GLIPH predicts HLA restriction and antigenic ligands, aiding in T cell response analysis for immunotherapy 108. GLIPH2, an advanced version, efficiently processes millions of TCRs, identifying specificity groups and epitopes. Applied to 19,044 TCRβ sequences from *M. tuberculosis*-infected individuals, GLIPH2 identified at least five PPE proteins as T-cell targets, enhancing pMHC-TCR pairing for complex pathogens 109. Notably, GIANA (Geometric Isometry-based TCR Alignment Algorithm) clusters TCR sequences by similarity with 600-fold greater efficiency than TCRdist, while maintaining clustering specificity. Capable of rapid querying across large reference cohorts, GIANA identifies candidate disease-specific receptors and advances TCR repertoire classification. By supporting pMHC-TCR pairing and TCR deorphanization, GIANA provides a foundation for a non-invasive, TCR-based diagnostic platform across cancer, infectious, and autoimmune diseases. TCRpcDist offers a 3D-based method for clustering TCRs by calculating similarities based on the physico-chemical properties of loop residues interacting with epitopes. This approach overcomes limitations of sequence-based techniques, particularly in data-scarce scenarios, and accurately identifies TCRs likely to bind the same epitopes. Validated to determine neoantigen and tumor-associated antigen specificities of orphan TILs in cancer patients, TCRpcDist advances TCR repertoire analysis and deorphanization for personalized cancer immunotherapy 110. Moreover, HeteroTCR, a supervised model based on Heterogeneous Graph Neural Networks, predicts peptide-TCR binding probabilities by capturing both within-type (TCR-TCR, peptide-peptide) similarities and between-type (peptide-TCR) interactions. Addressing limitations of existing models, HeteroTCR excels in predicting interactions for novel peptides and TCRs. Validation on independent and single-cell datasets confirms its accuracy and robustness, demonstrating enhanced pMHC-TCR pairing capabilities crucial for immunogenicity studies and TCR deorphanization 111.

Additionally, artificial intelligence (AI) is transforming the identification of tumor-reactive TCRs, distinguishing them from bystander TCRs and facilitating the development of personalized T cell therapies. Using high-throughput TCR cloning and single-cell RNA sequencing, the machine learning tool predicTCR distinguishes tumor-reactive TCRs from bystanders in an antigen-agnostic manner. PredicTCR significantly improves specificity and sensitivity, achieving a geometric mean accuracy of 0.74 versus 0.38 for traditional methods. This advancement enables rapid prioritization of tumor-reactive TCR clonotypes, streamlining the development of personalized T cell therapies 112. Importantly, TRTpred, an antigen-agnostic *in silico* predictor, outperforms existing tools across diverse tumor datasets, enabling detailed analysis of tumor-reactive TIL repertoires. Combined with high-avidity TCR prediction and clustering (MixTRTpred), TRTpred identifies clinically relevant TCRs, validated both *in vitro* and *in vivo*, and supports the inclusion of orphan and private tumor-reactive TCRs for patients lacking common tumor antigens. This approach accelerates the selection of viable TCRs for TIL or TCR T cell therapy, advancing personalized treatments for solid tumors 113.

### Multifunctional TCR screening platforms

Multifunctional TCR screening platforms are integrated systems designed for high-throughput identification and characterization of TCRs. These platforms merge advanced technologies like synthetic biology, microfluidics, and next-generation sequencing to efficiently identify TCRs specific to antigens on MHC molecules. They enable the generation of synthetic TCR libraries and the performance of functional assays to assess T cell responses. Additionally, many incorporate computational models to predict and analyze TCR-antigen interactions, improving antigen discovery essential for therapeutic developments. These platforms aim to accelerate the creation of targeted T-cell-based therapies, crucial for advancing precision medicine in oncology. Their comprehensive capabilities not only enhance the scope and accuracy of TCR screening but also establish these systems as indispensable in immunotherapy research.

For instance, DoubletSeeker, a high-throughput method, identifies ligand-receptor interactions by detecting cell doublets formed via specific membrane interactions. This approach captures paired TCR-pMHC information during complex library screenings, enabling precise identification of TCR-antigen interactions. DoubletSeeker uncovered mutant TCRs specific to the MART-1 epitope, advancing antigen discovery for immunotherapy development 114. Similarly, a high-throughput personalized TCR discovery pipeline enables the assembly of synthetic TCR libraries in a one-pot reaction, followed by pooled expression in reporter T cells and functional screening against patient-derived tumor cells. This method screens patient-derived TCRs for tumor specificity, identifying both MHC class I- and II-restricted TCRs targeting tumor-associated antigens, including neoantigens 115. Notably, a streamlined approach matches TCR sequences with cognate antigens through on-demand cloning, expression, and screening against candidate antigens. This system identifies viral- and neoantigen-specific TCRs, enabling rapid assessment of antigen specificity and functional avidity. Applied to melanoma and CLL, the method facilitates TCR discovery and aids in the development of T-cell-based immunotherapies 116. Interestingly, TCR-MAP is an antigen discovery method that uses a synthetic TCR circuit to activate sortase-mediated tagging of APCs presenting peptides on MHCs. Tagged APCs are purified for sequencing, enabling pooled screening of unknown TCR specificities against barcoded peptide libraries. This method captures both MHC class I- and class II-restricted TCR reactivities, identifying self, viral, and cancer-related targets with high sensitivity 117. TScan-II is a genome-scale CD4^+^ antigen discovery platform that integrates endogenous HLA-II processing in synthetic APCs with TCR signaling, enabling simultaneous screening of multiple HLAs and TCRs. Using human, virome, and mutagenesis libraries, it uncovers novel antigens and explores TCR specificity. TScan-II identified cancer-reactive and Sjögren's disease-specific antigens, highlighting its utility in both basic and translational research 118. Moreover, nanovials coated with peptide-MHC monomers capture and activate antigen-reactive T cells, accumulating secreted effector molecules like IFN-γ and granzyme B. Microfluidic single-cell sequencing recovers paired TCR αβ-chains from sorted cells. Oligo-barcoded nanovials and detection antibodies link TCRs to specific targets, enabling functional ranking. This approach expands the repertoire of functional TCRs, including rare cancer-specific TCRs 119.

## Clinical applications of TCR-T cell therapy

The clinical application of TCR T cell therapies targeting TAAs and TSAs is a critical development in precision oncology. These therapies are currently being evaluated for their ability to precisely target antigens predominantly expressed by tumor cells, optimizing therapeutic outcomes while minimizing unintended effects. The selectivity of TCRs for TAAs and TSAs enhances therapeutic efficacy, potentially transforming the management of advanced and refractory cancers. Advances in genomic profiling and TCR validation are expected to refine these therapies further, facilitating their broader integration into clinical practice.

### Clinical trials of TCR-T cell therapy targeting TAAs

The MAGE family encodes TAAs, which are highly expressed in various cancers but minimally in normal tissues. These antigens are prime targets for cancer immunotherapy, enabling the development of targeted vaccines and cellular therapies. For instance, TCR-T cell therapy targeting MAGE-A1 showed a manageable safety profile in a first-in-human trial involving patients with advanced solid tumors. IMA202, consisting of engineered autologous CD8^+^ T cells, demonstrated sustained biological activity, with detectable persistence in peripheral blood and tumor tissues. Notably, a substantial proportion of patients achieved stable disease, with some showing tumor shrinkage, underscoring the potential efficacy of this approach in solid tumor immunotherapy 120. Similarly, TCR-engineered T cells targeting MAGE-A10 demonstrated safety and therapeutic potential in a phase I trial for advanced non-small cell lung cancer. Patients received escalating doses of ADP-A2M10 SPEAR T cells, with observed responses ranging from stable disease to partial remission. While the trial confirmed the persistence of these cells in blood and tumor tissue, it was closed in favor of pursuing therapies targeting MAGE-A4 due to overlapping antigen expression 121. Afamitresgene autoleucel (TECELRA®) is an HLA-restricted autologous T cell therapy that targets MAGE A4 in HLA-A02-eligible patients with solid tumors, showing early and durable responses in patients with relapsed/refractory metastatic solid tumors. Notably, TECELRA® received FDA approval in the USA in August 2024 and is prescribed for adults with unresectable or metastatic synovial sarcoma who have previously undergone chemotherapy, following positive outcomes in clinical trials 122. In a phase 1 trial, the overall response rate was 24%, with 44% in synovial sarcoma patients. The therapy infiltrated tumors, triggered interferon-γ-driven immune responses, and showed an acceptable safety profile, with cytokine release syndrome being the most common adverse event 123. In a phase 2 trial, TECELRA® also showed promising efficacy and manageable safety in patients with advanced synovial sarcoma or myxoid round cell liposarcoma expressing HLA-A*02. In a heavily pre-treated cohort, an overall response rate of 37% was observed, with durable responses and manageable adverse events, such as cytokine release syndrome and cytopenias 124.

NY-ESO-1 is a cancer/testis antigen predominantly expressed in various tumors but rarely in normal tissues, making it a prominent target for cancer immunotherapy. Its distinct expression pattern supports the development of vaccines and TCR-based therapies to target cancer cells. A Phase 1 trial combining NY-ESO-1-specific TCR-engineered T-cell therapy with a lymph node-targeting nanoparticulate peptide vaccine demonstrated promising efficacy in treating refractory soft tissue sarcoma. This regimen, employing pullulan nanogel-loaded long peptide antigens, enhanced TCR-T cell accumulation and function in tumors, without lymphodepletion. Notable tumor shrinkage and prolonged TCR-T cell persistence were observed, suggesting a viable strategy for treating cold tumors 125. Importantly, autologous T lymphocytes engineered to express a TCR targeting NY-ESO-1 showed significant efficacy in a trial involving patients with advanced or recurrent synovial sarcoma. Despite a 50% objective response rate and manageable safety profile, adverse events were common, though serious complications such as cytokine release syndrome were effectively managed. This TCR T cell therapy offers a novel therapeutic option for tumors resistant to conventional treatments 126. Similarly, autologous T cells engineered with an affinity-enhanced TCR targeting the NY-ESO-1/LAGE1a peptide demonstrated significant therapeutic efficacy in metastatic synovial sarcoma. Treated patients exhibited durable responses and persistent T cell activity, with 50% achieving tumor shrinkage. The longevity and functionality of these T cells, characterized by a predominantly central memory phenotype, highlight their potential to sustain antitumor effects without signs of exhaustion, even under continuous antigen exposure 127.

Significantly, advancements in TCR engineered T cell therapies have shown promising results in targeting specific antigens associated with hematologic cancers, demonstrating their potential in overcoming resistance mechanisms and improving patient outcomes. For instance, in a phase 1 clinical trial, T cells engineered to express TCRs targeting the minor H antigen HA-1 were administered to patients with recurrent leukemia post-allogeneic stem cell transplantation. These HA-1 TCR-T cells, derived from disparate donors, expanded *in vivo* and were well-tolerated, showing preliminary signs of efficacy. Four patients achieved or maintained complete remissions, with one remaining in remission after two years 128. In addition, engineered T cells expressing a high-affinity Wilms' Tumor Antigen 1-specific TCR (TCRC4) were infused into patients with high-risk acute myeloid leukemia post- hematopoietic cell transplantation, achieving 100% relapse-free survival at 44 months, compared to 54% in a matched control group. Modified to minimize graft-versus-host disease, these TCR-T cells persisted long-term and exhibited multifunctional capabilities, suggesting a viable strategy for preventing AML recurrence 129. Notably, engineered T cells expressing TCRs targeted an HLA-A2-restricted Wilms' tumor antigen epitope in AML, but encountered resistance due to limited immunoproteasome activity. An alternative TCR that recognizes a proteasome-independent epitope effectively killed resistant AML and solid tumor lines *in vitro* and in NSG mice, suggesting a strategy to overcome proteasome-related immune evasion in AML 130.

### Clinical trials of TCR-T cell therapy targeting TSAs

Viral antigens serve as ideal targets for TCR T cell therapies as they are foreign proteins expressed bor cells infected with oncogenic viruses, providing highly specific and immunogenic targets. This specificity makes them excellent candidates for developing targeted immunotherapies with minimal risk of harming normal tissues. TCR-engineered T cells targeting cytomegalovirus (CMV) demonstrated safety and efficacy as a first-line pre-emptive therapy in a phase I trial for CMV reactivation post-haploidentical peripheral blood stem cell transplantation. The treatment resulted in rapid CMV clearance in most patients without the need for additional antiviral agents, with sustained T cell expansion and minimal adverse effects, highlighting its potential as a viable alternative to conventional antiviral therapies 121. Additionally, transiently functional TCR T cells, targeting hepatitis B virus antigens, demonstrated safety in a Phase I trial involving HCC patients post-liver transplant. These mRNA-electroporated T cells, specific to HBV-derived epitopes, showed minimal adverse effects and a tolerable profile, indicating a viable approach for addressing HBV-associated HCC recurrence 131. A phase 1/2 trial on TCR T-cell therapy for HPV-assocy tumiated epithelial cancers demonstrated preliminary efficacy and high tolerability. Patients received high doses of autologous T cells targeting HPV16 E6, leading to significant peripheral blood engraftment and objective tumor responses, including complete and partial regressions 132. Moreover, a novel TCR (10F04), identified from a metastatic cervical cancer patient who benefited from multiple antigens stimulating cellular therapy, has shown promise in targeting HPV18-positive tumors. This HLA-DRA/DRB1*09:01 restricted TCR redirects both CD4^+^ and CD8^+^ T cells to recognize and attack tumor cells effectively, demonstrating robust antitumor activity and safety *in vitro* and *in vivo* 133.

Neoantigens, unique peptides arising from tumor-specific mutations, are highly effective targets for TCR-engineered T cell therapies due to their exclusive expression in cancer cells and absence in normal tissues. Two TCRs specific to the KRAS-G12V mutant neoantigen presented by HLA-A*11:01 were identified. TCR-T cells constructed with these TCRs showed cytokine secretion and cytotoxicity against KRAS-G12V-expressing tumor cells. In preclinical models, these TCR-T cells demonstrated potent anti-tumor activity 134. In addition, an automated, good manufacturing practice-compliant process was developed for the scaled production of mutant NPM1 (dNPM1)-specific TCR-engineered T cells targeting AML. The optimized process shortened manufacturing time from 12 to 8 days, producing up to 5.5 billion CD8^+^ T cells with early memory phenotypes. These cells demonstrated specific AML killing *in vitro* and *in vivo*, supporting their use in an upcoming phase 1/2 clinical trial for NPM1-mutated AML 135. Interestingly, NeoScreen is a method that facilitates the sensitive identification of rare tumor neoantigens and their corresponding TCRs from tumor-infiltrating lymphocytes. T cells engineered with these specific TCRs, identified through NeoScreen, effectively mediate tumor regression in patient-derived xenograft models, demonstrating its utility in enhancing cancer immunotherapy 136. A high-throughput platform identified a panel of TCRs targeting a public neoantigen derived from a common PIK3CA mutation, presented by HLA-A*03:01. This neoantigen's immunogenicity arises from enhanced peptide/HLA complex stability. Structural analysis revealed that a lead TCR candidate binds the epitope with high specificity via an extended CDR3β loop. *In vivo*, TCR-engineered T cells targeting this neoantigen led to tumor regression in PIK3CA-mutant tumors, demonstrating the therapeutic potential of targeting public neoantigens in TCR T cell therapy 137.

In addition to its success in preclinical models, TCR T cell therapy has also shown promising results in clinical trials. CRISPR-Cas9 genome editing was utilized to replace endogenous TCRs with neoantigen-specific TCRs in T cells from patients with refractory solid tumors. This first-in-human trial demonstrated that engineered T cells could home to tumors and were associated with manageable side effects. Despite only stable disease or progression observed, the approach confirms the feasibility of precise TCR replacement for targeting mutational neoantigens in cancer therapy 138. In addition, a patient with metastatic pancreatic cancer received autologous T cells genetically engineered to express TCRs targeting the KRAS G12D mutation. The patient experienced a 72% reduction in tumor burden, and the response persisted at 6 months. Engineered T cells remained detectable, constituting over 2% of circulating T cells. This case demonstrates the potential of TCR T cell therapy to mediate significant tumor regression in KRAS-driven pancreatic cancer 139. A library of 39 TCRs targeting TP53 mutations, shared by 7.3% of solid tumor patients, demonstrated tumor-specific reactivity. In clinical trials, adoptive cell therapy with TILs yielded limited responses due to low frequencies of reactive, exhausted TILs. However, a patient treated with peripheral blood lymphocytes transduced with a TP53-specific TCR showed 55% tumor regression, indicating that targeting shared TP53 neoantigens holds promise for cancer treatment 140. In terms of adverse reactions, although TCR-T cell therapy has been linked to isolated, transient fevers in some patients, likely reflecting an initial immune activation as TCR-T cells target tumor antigens. This mild febrile response is typically manageable and does not appear to compromise therapeutic efficacy or patient safety.

### Challenges and prospects in TCR-based cancer immunotherapies

#### Manufacturing challenges and costs

While personalized T-cell therapy holds significant promise for treating various diseases, it faces substantial manufacturing challenges that impede broader application. These challenges include high production costs, complexities in scaling up production, intricate logistics, lengthy processing and testing times, and limited patient access. Specifically, the production of TCR-T products is both costly and complex, typically requiring 1-3 weeks 6, 141. This process entails several labor-intensive steps such as monocyte separation, T-cell activation for gene engineering susceptibility, amplification of gene-modified cells, and their subsequent harvesting and cryopreservation. These personalized therapies inherently preclude large-scale production, leading to intricate manufacturing processes and escalated costs 142. For example, the cost of a single infusion of therapies like axicabtagene ciloleucel or tisagenlecleucel can be as high as $373,000 and $475,000, respectively 143.

To address these significant challenges and improve the accessibility of TCR-T therapies, it is crucial to integrate more advanced manufacturing technologies. The adoption of automated bioreactors and closed-system cell processing units can streamline the complex steps involved in T-cell therapy production 144. Automation helps standardize processes such as monocyte separation, T-cell activation, and the amplification of gene-modified cells, thereby reducing human error and variability in production. Moreover, the implementation of continuous manufacturing strategies can further enhance the efficiency of these processes. By reducing batch-to-batch variability and shortening the lengthy timelines typically associated with personalized treatments, these strategies can significantly cut production costs and increase throughput. Implementing these advanced technologies in GMP-compliant facilitie is essential not only for maintaining product quality and safety but also for reducing overhead costs and enhancing scalability. By enhancing reproducibility and reducing both the time and expense associated with producing TCR-T therapies, these technological improvements have the potential to make these innovative treatments more accessible to a wider range of patients 145, 146. This broader accessibility is critical for the future of personalized medicine, as it promises to bring potent, targeted therapies to more individuals, ultimately improving outcomes in the treatment of complex diseases.

#### Dysfuction of administered T cells

Maintaining the persistence and activity of administered T cells is critical for effective anti-tumor responses in T cell immunotherapy. T cell exhaustion, stemming from repeated activation during chronic infection or tumor progression, alongside T cell anergy caused by over-activation of the TCR and strong co-inhibition, presents significant challenges that must be addressed 147. An effective strategy to enhance T cell persistence is non-myeloablative lymphodepleting chemotherapy, which administers specific chemotherapeutic agents before T cell injection. This approach reduces competition for crucial cytokines such as IL-7 and IL-15 between infused and endogenous T cells, enhancing their survival and function within the patient's body 148, 149. Furthermore, less differentiated T cell subsets, such as central memory T cells and stem cell memory T cells, demonstrate superior engraftment and persistence compared to more differentiated effector memory T cells and terminally differentiated effector cells 150. Techniques to enhance T cell efficacy include shortening T cell expansion time, employing less inductive cytokines, dedifferentiating induced pluripotent stem cells (iPSCs), or rejuvenating endogenous T cells to overcome dysfunction and exhaustion 151, 152. Enhancing TCR modification to improve T cell activity within the tumor microenvironment is also beneficial. This can be achieved by integrating co-stimulatory domains, co-expressing stimulatory CARs, or blocking inhibitory receptors 153. Genetic modifications, such as engineering T cells to self-secrete cytokines like IL-15 or IL-12, or using a constitutively activated IL-7 receptor, can stabilize cytokine signaling and enhance T cell persistence 143. Selective apoptosis to eliminate dysfunctional T cells helps maintain the proliferation of effective effector and memory cells. Bioengineering strategies, such as using thymus-like organ substances, growth factors, and cytokines like IL-21, support the restoration and maintenance of the thymic environment, reversing thymic involution. For instance, injecting allogeneic hematopoietic cells into a reconstituted thymus may rejuvenate the production of functional T cells 154. These strategies aim to boost T cell persistence and activity within the patient, thereby enhancing the effectiveness of immunotherapy. However, these approaches require further research and clinical trials for validation and optimization before widespread clinical application.

#### Antigen selection

The selection of appropriate tumor antigens as therapeutic targets is crucial in TCR-T cell therapy. The pool of safe and effective TAAs is limited, and the ability of tumor cells to downregulate or lose the expression of targeted antigens under immune pressure can significantly compromise the efficacy of TCR-T cell therapy 154. To address this challenge, several strategies have been proposed. One approach involves the use of high-throughput sequencing and computational biology techniques, such as HTS-IR, TraCeR, and single-cell TCR sequencing, which provide a systematic method for analyzing large datasets of TCR sequences and associated antigen information 155. These technologies enable the identification of novel TAAs and neoantigens, offering more precise and comprehensive targets for TCR-T cell therapies. Additionally, advanced techniques like flow cytometry, mass cytometry, and microfluidic technologies allow for the identification of tumor antigen-specific T cells within a patient's immune system, facilitating the development of personalized treatments tailored to the individual's specific tumor antigen profile. Moreover, genetic engineering technologies enable the introduction of multiple TCRs with distinct tumor specificities into a single population of T cells, creating polyclonal TCR-T cells 156. This polyclonal approach allows the T cells to target multiple antigens simultaneously, enhancing both the breadth and effectiveness of the treatment. By recognizing different tumor antigens, the therapy becomes more resilient to immune escape mechanisms, thereby broadening its scope and improving its specificity. This approach addresses the inherent complexities of antigen selection and significantly enhances the therapeutic potential of TCR-T cell therapy 157.

#### TCR optimization and functional expression

To fully harness the therapeutic potential of TCR-T cell therapy, one major challenge is ensuring that the TCRs bind with sufficient affinity to TAAs without causing off-target effects. Low-affinity TCRs may fail to generate a robust immune response, while overly high-affinity TCRs increase the risk of targeting healthy tissues. Another challenge involves ensuring proper surface expression of the engineered TCRs, as inefficient TCR expression or rapid internalization can limit the efficacy of TCR-T cells. Addressing these challenges is critical to improve the therapeutic effectiveness of TCR-T cell therapy.

One key strategy to improve the effectiveness of TCR-T cell therapy involves optimizing TCR affinity for specific antigens. This can be achieved through selective modifications of the CDRs, particularly in the CDR3 region of the TCR α and β chains, where amino acid substitutions can be introduced to fine-tune the affinity of the TCR for its target antigen 158. A structure-guided design process that incorporates both positive and negative design elements enables the detailed understanding of the three-dimensional structure of TCR-antigen interactions. This strategy allows for targeted mutations to either enhance or attenuate the binding interaction, optimizing the therapeutic response while minimizing potential off-target effects. In addition, optimizing the expression and function of the TCR protein is critical for enhancing TCR-T cell activity. This can be achieved by improving the pairing efficiency of the TCR α and β chains, utilizing optimized codon usage, and minimizing TCR glycosylation, which can otherwise hinder TCR surface expression and lead to internalization of transduced TCRs 154. Minimizing glycosylation can enhance the functional affinity of the TCR and improve its stability on the cell surface, thereby increasing the overall efficacy of the immune response.

#### TCR mismatch

The heterodimers formed through interactions within the TCR constant region by the TCR α/β chains might experience mismatch with the endogenous TCR α/β chains. This could, in turn, reduce the surface expression of the introduced TCR. Additionally, the mismatched TCRs may compete for the limited CD3 components for binding 159. More concerningly, these mismatches can create entirely novel TCRs, which have bypassed thymic selection and may unexpectedly target self-antigens 160. Therefore, the resolution of these mismatches is integral to preserving the efficacy of TCR-T cell therapy. Currently, the primary methods to enhance the expression level of introduced TCRs and to improve the matching or binding of the exogenous TCR α/β chains mainly involve modifying the TCR α/β chains. This is achieved by altering the nucleotide sequence of the TCR gene to ensure effective translation within host cells 161. For instance, replacing leucine and valine residues in the transmembrane region of the TCR alpha chain, and introducing part or all of the gene sequences of the mouse constant region into the human TCR, have been proven to enhance the stability and surface expression of the introduced TCR alpha chain 162, 163. Additionally, the strategy of introducing extra disulfide bonds in the TCR constant region can also enhance the stability and pairing efficiency of the introduced TCR 164, 165. For example, by introducing cysteine at Cα residue 48 and Cβ residue 57 to create a second stable disulfide bond, this improves the correct pairing of the introduced TCR. Furthermore, methods such as using a single-chain TCR, swapping two amino acids that mediate TCR α/β chain dimerization, or employing TCR/CD3 fusion products also hold promise in resolving mismatches in the TCR beta chain 166, 167.

## Conclusions

In conclusion, TCR-T cell therapy represents a major advancement in cancer immunotherapy, significantly enhancing the ability of T cells to selectively target and eliminate cancer cells. Despite substantial progress in TCR engineering and screening technologies, challenges related to the specificity, affinity, and safety of TCRs remain critical hurdles. The successful application of these therapies relies on precise and safe TCR selection. As research continues to evolve, addressing these challenges will be crucial to fully realizing the therapeutic potential of TCR-T cell therapy in cancer treatment.

## Figures and Tables

**Table 1:** Clinical trials of TCR-T in anti-tumor therapy

Cancer type	Target	Type	Results	HLA	Trial ID	Phase	Ref
Metastatic melanoma synovial cell sarcoma	MART-1 F5	TAA	\	\	NCT00923195	Phase 2	168
Metastatic melanoma	MART-1 F5	TAA	69% (9/13) of patients showed tumor regression	HLA-A*0201	NCT00910650	Phase 2	169
Vaginal cancer Cervical cancer Anal cancer Penile cancer Oropharyngeal cancer	HPV-16 E6	TSA	\	\	NCT02280811	Phase 1 Phase 2	NA
Cervical cancer Renal cancer Urothelial cancer Melanoma Breast cancer	MAGE-A3-DP4	TAA	\	\	NCT02111850	Phase 1 Phase 2	NA
Melanoma Meningioma Breast cancer NSCLC Hepatocellular cancer	NY-ESO-1	TAA	\	\	NCT01967823	NA	NA
Ovarian cancer	NY-ESO-1	TAA	\	\	NCT01567891	Phase 1 Phase 2	NA
Neoplasms	NY-ESO-1	TAA	\	\	NCT02992743	Phase 2	NA
NSCLC	NY-ESO-1	TAA	\	\	NCT02588612	Phase 1	NA
Metastatic cancer	p53	TAA	\	\	NCT00393029	Phase 2	NA
Progressive metastatic malignancies	p53	TAA	\	\	NCT00496860	Phase 1	NA
Neoplasms	NY-ESO-1	TAA	\	\	NCT01343043	Phase 1	NA
Solid tumor	MAGEA1	TAA	Of the 16 patients dosed, 11 (68.8%) patients had SD as their best overall response	HLA-A*02:01	NCT04639245 NCT05430555	Phase 1 Phase 2	120
Recurrent leukemia	HA-1	TAA	Patients achieved or maintained remission, with one lasting over 2 years	HLA-A*02:01	NCT03326921	Phase 1	128
HBV-related hepatocellular carcinoma	HBV	TSA	No CRS nor neurotoxicity	HLA-A02:01, HLA-A11:01	NCT02719782	Phase 1	131
Soft tissue sarcoma	NY-ESO-1	TAA	Treatment was well-tolerated; two had mild CRS. One showed tumor shrinkage lasting over two years.	HLA-A*02:01	JMA-IIA00346	Phase 1	125
Refractory solid tumors	Neoantigens	TSA	Five patients had stable disease and the other eleven had disease progression	HLA-A:02*01	NCT03970382	Phase 1	138
HPV16- positive epithelial cancer	HPV	TSA	All patients had high E6 TCR T cell engraftment. Two of 12 (17%) had tumor responses	HLA-A*02:01	\	Phase 1 Phase 2	132
B-Cell acute lymphoblastic leukemia	leukemia-associated antigens	TAA	Patients achieved complete remission 4 weeks	\	NCT03953599	Phase 1	170
Synovial sarcoma	NY-ESO-1	TAA	Tumor burden decreased after 4 weeks, with maximal responses in 4 patients after 3 months.	HLA-A*02:01	NCT01343043	Phase 1	127
Synovial Sarcoma	NY-ESO-1	TAA	All patients had adverse events, with 87.5% experiencing drug reactions, but no deaths occurred	HLA-A*02:01 HLA-A*02:06	NCT03250325	Phase 1 Phase 2	126
Sarcoma	MAGE-A4	TSA	Overall response rate was 37%	HLA-A*02	NCT03132922	Phase 2	124
Synovial sarcoma Ovarian cancer Head and neck cancer	MAGE-A4	TSA	All 38 patients had Grade ≥3 hematologic toxicities. CRS occurred in 55%. ORR was 24%, 44% for SS, and 9% for other cancers	HLA-A*02	NCT03132922	Phase 1	123

## Abbreviations

APC: Antigen-presenting Cell
CDR: Complementarity-determining Region
CEA: Carcinoembryonic Antigen
CGA: Cancer Germline Antigen
CMV: Cytomegalovirus
DC: Dendritic Cell
ERAAP: ER-associated Aminopeptidase
ITAM: Immunoreceptor Tyrosine-based Activation Motif
MHC: Major Histocompatibility Complex
MⅡCs: MHC class Ⅱ Compartments
NART: Neoantigen-reactive CD8^+^ T Cell
NGS: Next-generation Sequencing
TAA: Tumor-associated Antigens
TCR-T: T Cell Receptor-engineered T
TIL: Tumor-infiltrating Lymphocyte
Treg: Regulatory T Cell
TSA: Tumor-specific Antigens
